# An introduction to insulin use in type 2 diabetes mellitus

**DOI:** 10.4102/safp.v65i1.5702

**Published:** 2023-04-20

**Authors:** Ankia Coetzee

**Affiliations:** 1 Department of Medicine, Division of Endocrinology, Tygerberg Academic Hospital, Cape Town, South Africa; 2 Faculty of Medicine and Health Sciences, Stellenbosch University, Cape Town, South Africa

**Keywords:** insulin, type 2 diabetes, practical, multifactorial, basal

## Abstract

The benefits of the newer antidiabetic agents available for managing type 2 diabetes mellitus (T2DM) remain indisputable, but many patients will require insulin therapy in the disease course. Given the limited access to newer antidiabetic agents, insulin remains a standard treatment modality in T2DM in South Africa. Early, multifactorial intervention remains ideal, but glucose, blood pressure and cholesterol values remain above target in many countries. Barriers to achieving glucose control in South Africa include the healthcare provider’s being unfamiliar with the practicalities of insulin administration, initiation and titration. This article highlights these gaps and offers pragmatic solutions to overcome them.

## Introduction

When the famous physician Elliott Joslin first used insulin in diabetes mellitus (DM), he likened the effect to a biblical piece described as ‘a valley of dry bones rising, being clothed in flesh and restored to life’.^1^ While insulin treated hyperglycaemic comas effectively after its discovery in 1921, it very quickly became apparent that insulin in too large doses can lead to hypoglycaemic comas and death.^2^ To this day, insulin remains the cornerstone of type 1 diabetes mellitus (T1DM) and diabetic emergency treatment in all. As type 2 diabetes mellitus (T2DM) is a progressive disease, insulin therapy is ultimately required in its chronic management.

The limitations of a ‘glucocentric approach’ in T2DM management are well established. Currently, the main focus in T2DM treatment is the prevention and management of cardiovascular disease (CVD), heart failure (HF) and chronic kidney disease (CKD).^3^ While glycaemic control remains essential, sodium–glucose-transporter-2 (SGLT2) inhibitors or glucagon-like peptide-1 (GLP-1) agonists are increasingly gaining traction due to their beneficial risk-benefit profile. Most of these benefits seem to be over and above their antihyperglycaemic properties, with or without using other antidiabetic medication.^3,4^ Notwithstanding, hyperglycaemia remains a risk factor for adverse cardiovascular (CV) outcomes and early glycaemic control in T1DM and T2DM leads to better results, commonly referred to as the ‘legacy effect’.^5^ In South Africa, insulin remains the third most widely used antihyperglycaemic agent in T2DM.^6^ Therapeutic inertia accounts for many unmet glucose targets.^7^ Local barriers to DM management on a healthcare provider level include limited knowledge of insulin therapy.^8^ Insulin use may also be hindered by concerns over hypoglycaemia and weight gain.^9,10^ Thus, it is prudent that practitioners in resource-limited settings are familiar with the practical aspects of insulin therapy (initiation, intensification).

## The ‘ABC’ of type 2 diabetes mellitus

The landmark Steno-2 trial in T2DM patients with microalbuminuria highlighted the importance of early intensive multifactorial intervention. Benefits included a lower risk of CVD, progression to end-stage CKD, and the need for retinal photocoagulation. The dramatic reduction in adverse outcomes was achieved by addressing the ‘*ABC*’, where ‘A’ is for haemoglobin A1c (HbA1c), ‘B’ is for blood pressure, and ‘C’ is for cholesterol to target.^11^ The treatment included readily available drugs, for example, metformin, sulphonylurea ± insulin, angiotension-converting enzyme (ACE) inhibitors and angiotensin receptor blockers (ARBs), and statins ± fibrates. It is therefore conceivable that South African T2DM patient outcomes may improve if these factors are adequately addressed early in its course.

When it comes to HbA1c or ‘A’, the effects of intensive treatment with glucose-lowering therapy vary greatly from patient to patient, as does their susceptibility to side effects. The recommended HbA1c level of 7% still applies to many patients, although individualisation of glycaemic management is key.^12,13,14^

## Pathophysiology for the pragmatic

The pathogenesis of T2DM has been studied extensively, and our understanding continues to evolve. Here, we focus on some core concepts underpinning insulin therapy in chronic T2DM management.

### Normal glucose metabolism

Glucose concentrations are determined by the rate at which glucose enters and leaves the circulation. Normal glucose disposal occurs when adequate amounts of insulin are secreted to counterbalance the degree of physiological insulin resistance (IR) and carbohydrate ingestion.^15,16^ The energy required to maintain normoglycaemia during an overnight fast is provided by gluconeogenesis, which is partially suppressed by insulin.^17,18^ Glucose metabolism also follows a circadian rhythm characterised by glucose peaks during the early morning and lower values at night.^17^ In normoglycaemic conditions, fasting glucose does not increase because adequate amounts of insulin counterbalance the IR, suppressing hepatic gluconeogenesis. Similarly, in the prandial (eating) state, the amounts of mealtime insulin secreted are tailored for a given carbohydrate load. Overall, adequate insulin action prevents fasting hyperglycaemia and limits the mealtime glucose excursion in normoglycaemic individuals.

### Abnormalities in type 2 diabetes mellitus

At least eight pathophysiologic defects contribute to hyperglycaemia in T2DM, but IR and progressive β-cell failure represent the hallmark disturbances.^19,20^ Historically, T2DM was depicted as a state of increased IR, but it is crucial to note that the β-cell function is significantly reduced (±50%) by the time hyperglycaemia develops.^19,21^ Yet, early intervention may delay the progression of β-cell failure.^19,22^

**FIGURE 1:**
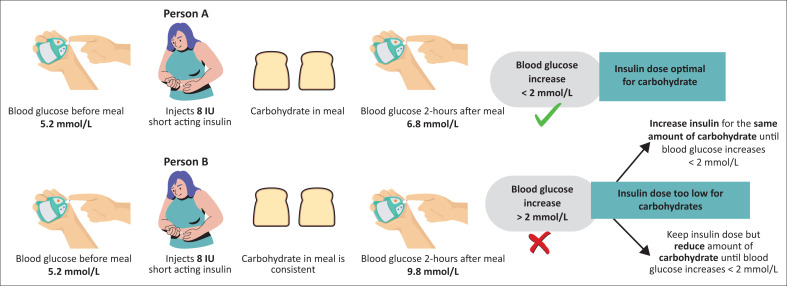
Matching the pre-meal short acting insulin dose with carbohydrate intake.

Paradoxically, in T2DM, the β-cell cannot secrete appropriate amounts of insulin to overcome a given IR, and the inability to suppress gluconeogenesis leads to fasting hyperglycaemia.^21,22^ In clinical practice, this abnormality is evident when patients with T2DM report an increase in glucose concentration overnight without ingesting any food or drink. Similarly, inadequate amounts of insulin for a given carbohydrate load during meals lead to postprandial hyperglycaemia^17,21^ (see Figure 1).

From a glucose perspective, insulin therapy in T2DM is therefore aimed at preventing excessive gluconeogenesis, that is, fasting hyperglycaemia with basal insulin and short (or ultra-short) acting insulin to limit the degree of postprandial glucose increases.^23^

There is no doubt that diabetes is one of the greatest natural disasters of our time. As with any natural disaster, it is important to remain informed and be prepared. As stated by Petra Nemcova:^24^

[*W*]e cannot stop natural disasters, but we can arm ourselves with knowledge …

## The bread and butter of insulin use in type 2 diabetes mellitus

In the following section, I will address some frequently encountered questions regarding insulin use in T2DM. It is by no means a comprehensive overview or guideline but a ‘snapshot’ of a few practical solutions and ‘tricks of the trade’ applicable in clinical practice.

Most importantly, the efficacy of insulin therapy depends on proper storage and injection technique. Correcting the insulin injection technique can lead to an improvement in glycaemic control and a reduction in the total daily dose of insulin.^25^ Sadly, access to structured diabetes education, including insulin administration, is limited in scope, content and consistency, leaving a sizeable gap in the public health sector in South Africa.^8^

Furthermore, T2DM management involves more than medication because it requires monitoring and limiting calorie intake as well as avoiding sedentary living.^26^ It has been demonstrated that this triad reduces the effect of insulin on weight gain by lowering insulin requirements and, consequently, reducing insulin’s anabolic effects.^27^

### Which patient with type 2 diabetes mellitus requires insulin?

Insulin should always be used in hyperglycaemic emergencies.^28^ Based on the expected HbA1c reduction of other antihyperglycaemic agents and the general target of ~7%, current guidelines endorse the initiation of insulin when HbA1c > 10% even at diabetes diagnosis, as the use of oral agents alone is unlikely to achieve targets.^28^ The early introduction of insulin should also be considered if:

- There is ongoing catabolism (weight loss) ± symptomatic hyperglycaemia.
- Blood glucose levels (≥ 16.7mmol/L) are very high.

### Should insulin be continued lifelong?

Hyperglycaemia paradoxically impacts insulin secretion by causing temporary β-cell ‘paralysis’, a phenomenon known as glucotoxicity.^29^ Early insulin can help overcome the glucotoxicity effects of hyperglycaemia by preserving β-cell mass and function.^29^ Therefore, depending on the duration ± degree of β-cell failure, exogenous insulin can successfully be discontinued in many patients with T2DM by addressing glucotoxicity and optimising lifestyle and other antihyperglycaemic agents.^30^

### What is the role of basal insulin in type 2 diabetes mellitus?

Basal insulin is primarily aimed at suppressing hepatic gluconeogenesis to prevent fasting hyperglycaemia.^3,31^ A wide variety of insulin preparations are available. Basal insulin analogues have the advantage of 24-h coverage and an action profile with low variability compared to human natural protamine Hagedorn (NPH) insulin. Natural protamine Hagedorn insulin may require twice-daily dosing and is associated with more hypoglycaemia than the basal analogues due to a ‘peak’ effect ~4 h – 6 h after injection.^31^ This peak is also utilised to limit the number of insulin injections by ‘standing in’ for a mealtime short-acting insulin injection. With NPH insulin and first-generation basal insulin analogues (Glargine-100 and detemir), the preferred timing of injection is bedtime.^32^ Second-generation basal insulin analogues (Degludec and Glargine-300) are ‘peak-less’ and may be administered at any but the same time each day. Glargine also has lower inter-subject variability compared to NPH.^33^

### How do I initiate and titrate basal insulin?

Basal insulin is usually initiated at 10 IU/day or 0.1 IU – 0.2 IU/kg. Titration in doses of 2–4 units should be performed once to twice a week until target fasting glucose values are achieved (5.6 mmol/L – 7.8 mmol/l are reasonable goals for most adults).^31^ In certain countries, patient-led titration is as (or equally) effective as physician-led titration.^34^ Still, it requires frequent self-monitoring of blood glucose, good insight and self-management ability.^32^ In practice, the ideal basal dose should allow a patient with T2DM to fast for 24 h without hypo or hyperglycaemia.

### What is meant by the term ‘overbasalisation’?

Overbasalisation is when the titration of basal insulin is beyond an appropriate dose. Basal insulin efficacy plateaus at doses > 0.5 units/kg per day. Therefore, the American Diabetes Association guidelines recommend considering additional treatment, such as mealtime short-acting insulin for post-meal coverage if glucose targets are not met.^12^ In insulin-sensitive patients, overbasalisation may manifest with paradoxically elevated fasting glucose resulting from preceding nocturnal hypoglycaemia. If this occurs, basal insulin doses should be reduced. Defensive eating, where patients have to adjust their diet by eating more to prevent hypoglycaemia, indicates too large respective insulin doses.

### The glucose targets are not met with basal insulin. What next?

There is more than one way to proceed with intensification, including adding a GLP-1 agonist.^35,36,37^ Options when using insulin are:

- The ‘step-up’ regimen adds one rapid or short-acting insulin injection before the main meal. This addresses the meal with the greatest glucose elevation by adding a short or ultra-short-acting insulin. Usually, the post-breakfast excursion is the highest, but it could also be the meal with the highest carbohydrate content. If the HbA1c target is not met, progress to the remaining meals by adding insulin sequentially towards a basal-bolus regimen (where each meal is covered with bolus insulin).
- Premixed insulin (e.g., NPH/regular 70/30, other formulations, e.g. 75/25 also available) is another option. This regimen allows less flexibility in dosing compared to a basal-bolus regimen (with a few exceptions) but has the advantage of fewer injections.

### What insulin regimen to use?

The management plan should be tailored to accommodate the patient’s daily activities, conditions and socioeconomic circumstances and not be a one-size-fits-all approach.^38^ In South Africa, many people with DM do not have access to three meals per day.^39^ Food insecurity is a risk factor for hypoglycaemia, subsequent hospitalisations and elevated HbA1c. The critical aspect is that basal insulin should be continued to prevent gluconeogenesis ± ketogenesis. Mealtime short-acting insulin is not required when not eating. In practice, basal insulin is optimised, and short-acting insulin is given only when a meal is available. The risk of hypoglycaemia increases when using insulin premix because basal insulin is inevitably omitted if the (mealtime) prandial insulin is not given.^40^

### When do I stop sulphonylureas?

Sulphonylureas are still the second most used oral agent in developing countries and resource-restrained environments after metformin.^41^ Diabetes results in a progressive decline in β-cell function.^42^ Sulphonylureas lower blood glucose by stimulating β-cell function and secrete more endogenous insulin. When the therapeutic regimen requires escalation beyond basal insulin, endogenous insulin secretion is unlikely to contribute significantly, with a few exceptions. The recommendation to discontinue the sulphonylurea (glyburide) but not metformin after insulin combination therapy is started is based on some patients’ inability to adequately titrate the dose of bedtime NPH because of hypoglycaemia.^43,44^ It has been proposed that sulphonylureas can be continued for meals not covered by pre-prandial insulin using basal-bolus regimens. Hypoglycaemia and weight gain are two of the most common and clinically significant side effects of combining sulphonylureas with insulin.^45^

### When is pre-meal insulin dose optimal?

**FIGURE 2:**
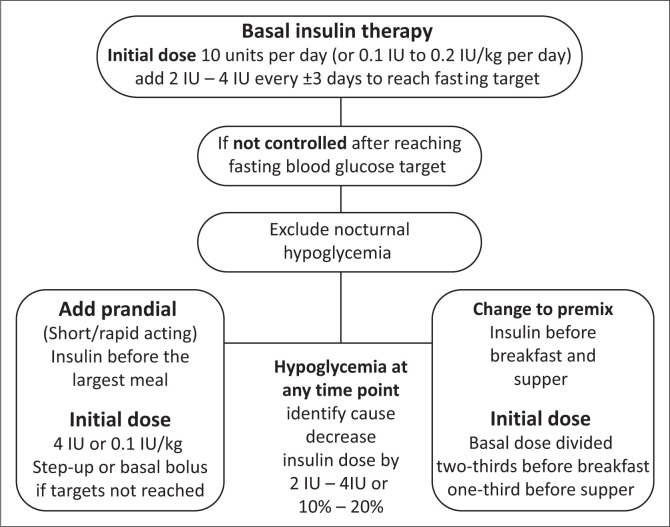
Insulin initiation based on American Diabetes Association guidelines.

The optimal dose differs between people, depending on many factors including the carbohydrate content of the meal and IR. A critical aspect is the relationship between pre-meal insulin and the carbohydrate content of meals.^46,47^ If pre-meal insulin doses are fixed, that is, 8 IU short-acting insulin administered before breakfast and 2-h post-meal (pre-lunch) glucose increases less than ±2 mmol/L; the respective insulin dose effectively counterbalances the meal’s carbohydrates for that person.^48,49^ Therefore, if the carbohydrate intake increases and the same insulin dose is given, it will not have the same effect. Options are to adjust the short-acting insulin at each meal, based on the carbohydrates, or to keep the carbohydrate content consistent for a specific insulin dose. Insulin adjustment is not an option with most premix insulins, because the dose of the intermediate component will also be altered and therefore carbohydrate consistency is essential. In my clinical practice, most patients prefer carbohydrate consistency over insulin adjustments. To allow variety in the diet, patients must work with a dietician ± diabetes educator to teach them carbohydrate exchanges, that is, different food with similar carbohydrate content.^48^ For example, one slice of bread is equivalent to three cups of popcorn, half a cup of cooked oatmeal, one cup of pumpkin and a small apple, orange or nectarine. Note that pre-meal insulin dose adjustment is not an option in a patient using premixed insulins (with a few exceptions) because the dose of long-acting will be affected, inevitably. Figure 1 illustrates the prandial adjustment, while Figure 2 illustrates the insulin adjustment when starting and when hypoglycaemia occurs.

Another essential point is the different onset of actions of short-acting insulin preparations. Ultra-short-acting insulins can be administered at mealtime, and some preparations require that it is injected 15 min – 30 min before the meal. Incorrect administration timing may lead to either hypo or hyperglycaemia, even when the amount of insulin effectively ‘matches’ the carbohydrates.

## Conclusion

Effective multifactorial treatment addressing the particular defects in an individual with T2DM will require early intervention and various drugs in combination. Given the limited access to newer antidiabetic agents, insulin remains a standard treatment modality in T2DM in South Africa. Early insulin initiation, optimisation and patient empowerment can help to manage T2DM effectively but will require professional education and upskilling in the practical management of DM. Patient empowerment with structured diabetes self-management education is vital in T2DM care, but access is limited in South Africa.

## References

[1] Sanders LJ. From Thebes to Toronto and the 21st Century: An incredible journey. Diabetes Spectr. 2002;15(1):56–60. 10.2337/diaspect.15.1.56

[2] Banting FG, Best CH, Collip JB, Macleod JJR, Noble EC. The effect of pancreatic extract (insulin) on normal rabbits. Am J Physiol-Leg Content. 1922;62(1):162–176. 10.1152/ajplegacy.1922.62.1.162

[3] American Diabetes Association Professional Practice Committee. 10. Cardiovascular disease and risk management: *Standards of medical care in diabetes – 2022*. Diabetes Care. 2022;45(suppl_1):S144–S174. 10.2337/dc22-S010 34964815

[4] Marx N, Davies MJ, Grant PJ, Mathieu C, Petrie JR, Cosentino F, et al. Guideline recommendations and the positioning of newer drugs in type 2 diabetes care. Lancet Diabetes Endocrinol. 2021;9(1):46–52. 10.1016/S2213-8587(20)30343-0 33159841 PMC12140926

[5] Holman RR, Paul SK, Bethel MA, Matthews DR, Neil HAW. 10-year follow-up of intensive glucose control in type 2 diabetes. N Engl J Med. 2008;359(15): 1577–1589. 10.1056/NEJMoa0806470 18784090

[6] Webb EM, Rheeder P, Van Zyl DG. Diabetes care and complications in primary care in the Tshwane district of South Africa. Prim Care Diabetes. 2015;9(2):147–154. 10.1016/j.pcd.2014.05.002 24933340

[7] Khunti S, Davies MJ, Khunti K. Clinical inertia in the management of type 2 diabetes mellitus: A focused literature review. Br J Diabetes. 2015;15(2):65. 10.15277/bjdvd.2015.019

[8] Parker N, Coetzee A, Van Wyk L, Conradie M. Practical aspects of insulin administration: What the healthcare provider knows. J Endocrinol Metab Diabetes South Afr. 2022;27(3):100–107. 10.1080/16089677.2022.2057692

[9] Current state and principles of basal insulin therapy in Type 2 Diabetes. J Clin Med Res. 2022;14(1):8–21. 10.14740/jocmr4660 35211212 PMC8827224

[10] Weir GC, Jameson JL, Groot LJD. Endocrinology adult and pediatric: Diabetes mellitus and obesity e-book. Elsevier Health Sciences, 2013; p. 542.

[11] Pedersen O, Gæde P. Intensified multifactorial intervention and cardiovascular outcome in type 2 diabetes: The Steno-2 study. Metabolism. 2003;52(suppl 1): 19–23. 10.1016/S0026-0495(03)00213-0 12939735

[12] American Diabetes Association. Professional practice committee: *Standards of Medical Care in Diabetes – 2022*. Diabetes Care. 2022;45(suppl_1):S3. 10.2337/dc22-Sppc

[13] Christiaens A, Henrard S, Zerah L, Dalleur O, Bourdel-Marchasson I, Boland B. Individualisation of glycaemic management in older people with type 2 diabetes: A systematic review of clinical practice guidelines recommendations. Age Ageing. 2021;50(6):1935–1942. 10.1093/ageing/afab157 34379732

[14] Webb D. 2017 SEMDSA diabetes management guidelines. South Afr J Diabetes Vasc Dis. 2018;15(1):37–40.

[15] Woerle HJ, Meyer C, Dostou JM, Gosmanov NR, Islam N, Popa E, et al. Pathways for glucose disposal after meal ingestion in humans. Am J Physiol-Endocrinol Metab. 2003;284(4):E716–E725. 10.1152/ajpendo.00365.2002 12475753

[16] Vlachos D, Malisova S, Lindberg FA, Karaniki G. Glycemic Index (GI) or Glycemic Load (GL) and dietary interventions for optimizing postprandial hyperglycemia in patients with T2 diabetes: A Review. Nutrients. 2020;12(6):1561. 10.3390/nu12061561 32471238 PMC7352659

[17] Radziuk J, Pye S. Diurnal rhythm in endogenous glucose production is a major contributor to fasting hyperglycaemia in type 2 diabetes. Suprachiasmatic deficit or limit cycle behaviour? Diabetologia. 2006;49(7):1619–1628. 10.1007/s00125-006-0273-9 16752180

[18] Radziuk J. Hepatic glycogen in humans. I. Direct formation after oral and intravenous glucose or after a 24-h fast. Am J Physiol-Endocrinol Metab. 1989;257(2):E145–E157. 10.1152/ajpendo.1989.257.2.E145 2669511

[19] DeFronzo RA. From the triumvirate to the ominous octet: A new paradigm for the treatment of type 2 diabetes mellitus. Diabetes. 2009;58(4):773–795. 10.2337/db09-9028 19336687 PMC2661582

[20] Abdul-Ghani M, DeFronzo RA. Personalized approach for type 2 diabetes pharmacotherapy: Where are we and where do we need to be?. Expert Opin Pharmacother. 2021;22(16):2113–2125. https://doi.org/10.1080/14656566.2021. 1967319 34435523 10.1080/14656566.2021.1967319

[21] Bock G, Chittilapilly E, Basu R, Toffolo G, Cobelli C, Chandramouli V, et al. Contribution of hepatic and extrahepatic insulin resistance to the pathogenesis of impaired fasting glucose. Diabetes. 2007;56(6):1703–1711. 10.1080/14656566.2021.1967319 17384334

[22] Kahn SE. The importance of β-cell failure in the development and progression of type 2 diabetes. J Clin Endocrinol Metab. 2001;86(9):4047–4058. 10.1210/jcem.86.9.7713 11549624

[23] Del Prato S, Tiengo A. The importance of first-phase insulin secretion: Implications for the therapy of type 2 diabetes mellitus. Diabetes Metab Res Rev. 2001;17(3):164–174. 10.1002/dmrr.198 11424229

[24] Nemcova P. Petra Nemcova quotes [page on the Internet]. BrainyQuote. [cited n.d.]. Available from: https://www.brainyquote.com/quotes/petra_nemcova_426837.

[25] Ichikawa M, Akiyama T, Tsujimoto Y, Anan K, Yamakawa T, Terauchi Y. Efficacy of injection technique education in diabetes with lipohypertrophy: A systematic review and meta-analysis protocol v3 [homepage on the Internet]. 2021 [cited 2022 Dec 28]. Available from: https://www.protocols.io/view/efficacy-of-injection-technique-education-in-diabe-btiinkce 10.1136/bmjopen-2021-055529 PMC8905878 35256444

[26] Wing RR, Blair EH, Bononi P, Marcus MD, Watanabe R, Bergman RN. Caloric restriction per se is a significant factor in improvements in glycemic control and insulin sensitivity during weight loss in obese NIDDM patients. Diabetes Care. 1994;17(1):30–36. 10.2337/diacare.17.1.30 8112186

[27] Sampath Kumar A, Maiya AG, Shastry BA, Vaishali K, Ravishankar N, Hazari A, et al. Exercise and insulin resistance in type 2 diabetes mellitus: A systematic review and meta-analysis. Ann Phys Rehabil Med. 2019;62(2):98–103. 10.1016/j.rehab.2018.11.001 30553010

[28] American Diabetes Association. 2. Classification and diagnosis of diabetes: Standards of medical care in diabetes – 2020. Diabetes Care. 2019;43 (suppl_1):S14–S31. 10.2337/dc20-S002 31862745

[29] Campos C. Chronic hyperglycemia and glucose toxicity: Pathology and clinical sequelae. Postgrad Med. 2012;124(6):90–97. 10.3810/pgm.2012.11.2615 23322142

[30] Robertson RP, Harmon J, Tran POT, Poitout V. β-cell glucose toxicity, lipotoxicity, and chronic oxidative stress in type 2 diabetes. Diabetes. 2004;53(suppl_1): S119–S124. 10.3810/pgm.2012.11.2615 14749276

[31] Bajaj S, Das AK, Kalra S, Sahay R, Saboo B, Das S, et al. BE-SMART (Basal Early Strategies to Maximize HbA1c Reduction with Oral Therapy): Expert opinion. Diabetes Ther. 2019;10(4):1189–1204. 10.3810/pgm.2012.11.2615 31102253 PMC6612329

[32] Mehta R, Goldenberg R, Katselnik D, Kuritzky L. Practical guidance on the initiation, titration, and switching of basal insulins: A narrative review for primary care. Ann Med. 2021;53(1):999–1010. 10.1080/07853890.2021.1925148 PMC8231382 34165382

[33] Rosenstock J, Dailey G, Massi-Benedetti M, Fritsche A, Lin Z, Salzman A. Reduced hypoglycemia risk with insulin glargine. Diabetes Care. 2005;28(4):950–955. 10.2337/diacare.28.4.950 15793205

[34] Blonde L, Merilainen M, Karwe V, Raskin P, TITRATE^TM^ study group. Patient-directed titration for achieving glycaemic goals using a once-daily basal insulin analogue: An assessment of two different fasting plasma glucose targets - the TITRATE^TM^ study. Diabetes Obes Metab. 2009;11(6):623–631. 10.1111/j.1463-1326.2009.01060.x 19515182

[35] Raccah D. Options for the intensification of insulin therapy when basal insulin is not enough in type 2 diabetes mellitus. Diabetes Obes Metab. 2008;10(s2):76–82. 10.1111/j.1463-1326.2008.00846.x 18577159

[36] Meece J. Basal insulin intensification in patients with type 2 diabetes: A review. Diabetes Ther. 2018;9(3):877–890. 10.1007/s13300-018-0395-3 29574634 PMC5984906

[37] Holman RR, Thorne KI, Farmer AJ, Davies MJ, Keenan JF, Paul S, et al. Addition of biphasic, prandial, or basal insulin to oral therapy in type 2 diabetes. N Engl J Med. 2007;357(17):1716–1730. 10.1056/NEJMoa075392 17890232

[38] Wu T, Betty B, Downie M, Khanolkar M, Kilov G, Orr-Walker B, et al. Practical guidance on the use of premix insulin analogs in initiating, intensifying, or switching insulin regimens in type 2 diabetes. Diabetes Ther. 2015;6(3):273–287. 10.1007/s13300-015-0116-0 26104878 PMC4575300

[39] Hunter-Adams J, Battersby J, Oni T. Food insecurity in relation to obesity in peri-urban Cape Town, South Africa: Implications for diet-related non-communicable disease. Appetite. 2019;137:244–249. 10.1016/j.appet.2019.03.012 30872143

[40] Gucciardi E, Vahabi M, Norris N, Del Monte JP, Farnum C. The intersection between food insecurity and diabetes: A review. Curr Nutr Rep. 2014;3(4): 324–332. 10.1016/j.appet.2019.03.012 25383254 PMC4218969

[41] Mohan V, Saboo B, Khader J, Modi KD, Jindal S, Wangnoo SK, et al. Position of sulfonylureas in the current ERA: Review of national and international guidelines. Clin Med Insights Endocrinol Diabetes. 2022;15:117955142210746. 10.1016/j.appet.2019.03.012 PMC8854230 35185350

[42] Mahler RJ, Adler ML. Type 2 diabetes mellitus: Update on diagnosis, pathophysiology, and Treatment. J Clin Endocrinol Metab. 1999;84(4):1165–1171. 10.1210/jcem.84.4.5612 10199747

[43] Kalra S, Bahendeka S, Sahay R, Ghosh S, Md F, Orabi A, et al. Consensus recommendations on sulfonylurea and sulfonylurea combinations in the management of Type 2 diabetes mellitus – International Task Force. Indian J Endocrinol Metab. 2018;22(1):132. 10.4103/ijem.IJEM_556_17 29535952 PMC5838894

[44] Janka HU, Plewe G, Riddle MC, Kliebe-Frisch C, Schweitzer MA, Yki-Järvinen H. Comparison of basal insulin added to oral agents versus twice-daily premixed insulin as initial insulin therapy for type 2 diabetes. Diabetes Care. 2005;28(2):254–259. 10.2337/diacare.28.2.254 15677775

[45] The right place for Sulphonylureas today: Part of ‘Review the series: Implications of recent CVOTs in Type 2 diabetes mellitus’. Diabetes Res Clin Pract. 2019;157:107836. 10.1016/j.diabres.2019.107836 31479704

[46] Basu A, Basu R. Insulin:Carbohydrate Ratio – Part of the story. Diabetes Technol Ther. 2015;17(12):851–853. 10.1016/j.diabres.2019.107836 26618719 PMC4677107

[47] Jenkins DJ, Wolever TM, Taylor RH, Barker H, Fielden H, Baldwin JM, et al. Glycemic index of foods: A physiological basis for carbohydrate exchange. Am J Clin Nutr. 1981;34(3):362–366. 10.1016/j.diabres.2019.107836 6259925

[48] Kulkarni KD. Carbohydrate counting: A practical meal-planning option for people with diabetes. Clin Diabetes. 2005;23(3):120–122. 10.2337/diaclin.23.3.120

[49] Vespasiani G, Rossi MC. Diabetes interactive diary: A mobile phone-based telemedicine system for carbohydrate counting and bolus calculator. In: Bruttomesso D, Grassi G, editors. Frontiers in diabetes [homepage on the Internet]. S. Karger AG; 2015 [cited 2022 Dec 28]. p. 226–235. Available from: https://www.karger.com/Article/FullText/363519.

